# Vitamin E As a Potential Interventional Treatment for Metabolic Syndrome: Evidence from Animal and Human Studies

**DOI:** 10.3389/fphar.2017.00444

**Published:** 2017-07-05

**Authors:** Sok Kuan Wong, Kok-Yong Chin, Farihah Hj Suhaimi, Fairus Ahmad, Soelaiman Ima-Nirwana

**Affiliations:** ^1^Department of Pharmacology, Faculty of Medicine, Universiti Kebangsaan MalaysiaCheras, Malaysia; ^2^Department of Anatomy, Faculty of Medicine, Universiti Kebangsaan MalaysiaCheras, Malaysia

**Keywords:** vitamin E, tocopherol, tocotrienol, metabolic syndrome, obesity, hyperglycemia, hypertension, dyslipidemia

## Abstract

A constellation of medical conditions inclusive of central obesity, hyperglycemia, hypertension, and dyslipidemia is known as metabolic syndrome (MetS). The safest option in curtailing the progression of MetS is through maintaining a healthy lifestyle, which by itself, is a long-term commitment entailing much determination. A combination of pharmacological and non-pharmacological approach, as well as lifestyle modification is a more holistic alternative in the management of MetS. Vitamin E has been revealed to possess anti-oxidative, anti-inflammatory, anti-obesity, anti-hyperglycemic, anti-hypertensive and anti-hypercholesterolemic properties. The pathways regulated by vitamin E are critical in the development of MetS and its components. Therefore, we postulate that vitamin E may exert some health benefits on MetS patients. This review intends to summarize the evidence in animal and human studies on the effects of vitamin E and articulate the contrasting potential of tocopherol (TF) and tocotrienol (T3) in preventing the medical conditions associated with MetS. As a conclusion, this review suggests that vitamin E may be a promising agent for attenuating MetS.

## Introduction

Metabolic syndrome (MetS) is characterized by the fulfillment of at least three or more health conditions, including central obesity, hyperglycemia, hypertension, and dyslipidemia (Alberti et al., 2009). MetS is a major socioeconomic challenge because it is associated with diseases, especially of the cardiovascular system, that reduces quality of life and incurs significant healthcare cost (Boudreau et al., 2009; Johnson et al., 2015). The incidence of MetS is predicted to escalate rapidly in future due to the alarming rise of obesity among adult population (Han and Lean, 2016). The development of MetS is closely related with sedentary lifestyle and unhealthy eating behavior (O'Neill and O'Driscoll, 2015). Lifestyle modification through adopting a recommended diet treatment plan with exercise is the safest long-term strategy for MetS (Kaur, 2014). Patients with MetS are encouraged to avoid food and beverages with high sugar (sucrose and fructose), fat, and carbohydrate contents. All these seemingly essential nutrients are harmful in large quantities and likely to cause MetS (Wong et al., 2016a). Alternatively, pharmacological therapy (appetite suppressants or nutrient absorption inhibitor) and surgical consideration (bariatric surgery) are applicable when the outcome of lifestyle modification is not positive or for individuals with comorbid obesity (Cornier et al., 2008; Karmali et al., 2010; Wong et al., 2016b). In addition to the above measures, the development of new pharmacological agents as therapy for MetS is necessary.

Vitamin E, first identified as an essential nutrient for reproduction in 1922 (Evans and Bishop, 1922; Brigelius-Flohe and Traber, 1999), is a lipid-soluble vitamin existing in two major subgroups: tocopherol (TF) and tocotrienol (T3). They are made up of four distinct analogs respectively: alpha (α), beta (β), gamma (γ), and delta (δ), differentiated by the location of methyl groups on the chromanol nucleus. Tocopherols are compounds with the long saturated (phytyl) tails and T3 are compounds with the short unsaturated (farnesyl) tails. Vitamin E (particularly αTF) is ubiquitously found in food such as vegetable oils, palm oil, rice bran, wheat germ, safflower, olives, soy beans, barley, nuts and grains. Meanwhile, the three most abundant sources of T3 are rice bran (50%), palm (75%), and annatto (99.9%) (Aggarwal et al., 2010). Recently, interest in the health benefits of vitamin E increased rapidly as emerging evidence on the anti-obesity (Zhao et al., 2015), anti-hypercholesterolemic (Zaiden et al., 2010), anti-diabetic (Budin et al., 2009; Matough et al., 2014), and anti-hypertensive (Newaz et al., 2003) effects of vitamin E has been reported. Both TF and T3 have been demonstrated to improve metabolic abnormalities in animal and human studies with varying levels of biological activity.

In view of the beneficial properties of vitamin E in treating the medical conditions associated with MetS, it would be interesting to investigate the possibility of vitamin E for prophylaxis or treatment of MetS. In this review, evidence on the biological activities of vitamin E in preventing the clinical features of MetS in animal and human studies is discussed in detail. The comparison between the effects of TF and T3 is also discussed. We hope this review will provide information to the readers on the potential of vitamin E as a treatment modality for MetS.

## Effects of vitamin E on MetS features in animal models

There is an impressive array of laboratory studies indicating directly or indirectly the potential influences of vitamin E on MetS using laboratory animal models. Animals serve as appropriate experimental models to solve the difficulties in evaluating the pathophysiology of MetS in humans (Wong et al., 2016a). The effects of vitamin E in obese (Table 1), diabetic (Table 2), hyperlipidemic (Tables 3A,B), hypertensive (Table 4), and MetS (Table 5) animal models have been summarized.

**Table 1:** The effects of vitamin E on metabolic parameters in obese animal models.

**Researcher (Year)**	**Animal model**	**Types of diet/induction**	**Treatment and dose**	**Mode**	**Duration Weeks**	**Changes in metabolic parameters**		
						**Obesity**	**Hyperglycemia**	**Dyslipidemia**
Zhao et al., 2015	Male C57BL/6J mice	High-fat diet	γT3 (10, 50 mg/kg)	Oral	4	↓	↓	–
Alcala et al., 2015	Male C57BL/6J mice	High-fat (45%) diet	αTF (150 mg/kg, twice a week)	Oral	28	↔	↓	↓
Hasty et al., 2007	C57BL/6 mice (ob/ob; LDLr^−/−^)	Western type high-fat (42%) diet	Vitamin E (2,000 IU/kg diet)	Oral	12	↔	–	↔

**Table 2:** The effects of vitamin E on metabolic parameters in diabetic animal models.

**Researcher (Year)**	**Animal model**	**Types of diet/induction**	**Treatment and dose**	**Mode**	**Duration Weeks**	**Changes in metabolic parameters**			
						**Obesity**	**Hyperglycemia**	**Hypertension**	**Dyslipidemia**
Chou et al., 2009	Male Wistar rats	STZ and nicotinamide	γT3 (6 g/kg diet)	Oral	5	↔	↓	–	↓
Kuhad et al., 2009	Male Wistar rats	STZ	T3 (25, 50, 100 mg/kg/day)	Oral	10	↑	↓	–	–
Kuhad and Chopra, 2009a,b	Male Wistar rats	STZ	T3 (25, 50, 100 mg/kg/day)	Oral	4	↑	↓	↓	–
			αTF (100 mg/kg/day)			↑	↓	↓	–
Budin et al., 2009	Male Sprague-Dawley rats	STZ	TRF (200 mg/kg/day)	Oral	8	–	↓	–	↓
Matough et al., 2014	Male Sprague-Dawley rats	STZ	TRF (200 mg/kg/day)	Oral	4	–	↓	–	–
Fang et al., 2010	Male C57BLKS/J-*Lepr Db/Db* mice	–	Palm TRF (50 mg/kg/day)	Oral	2	–	↓	–	–
Siddiqui et al., 2010	Male Wistar rats	STZ	Rice bran TRF (200 mg/kg/day)	Oral	8	–	↓	–	–
			Palm TRF (200 mg/kg/day)						
Siddiqui et al., 2013	Male Wistar rats	High fat diet and STZ	Rice bran TRF (400 mg/kg/day)	Oral	16	–	↓	–	↓
			Palm TRF (200 mg/kg/day)						

**Table 3:** The effects of vitamin E on metabolic parameters in hyperlipidemic animal models.

**Researcher (Year)**	**Animal model**	**Types of diet/induction**	**Treatment and dose**	**Mode**	**Duration**	**Changes in metabolic parameters**		
						**Obesity**	**Hyperglycemia**	**Dyslipidemia**
**(A) FEMALE ANIMALS**								
Qureshi and Peterson, 2001	White Leghorn female chickens	Corn-soybean diet	Rice bran TRF (50 ppm) + lovastatin (50 ppm)	Oral	4 weeks	–	–	↓
Yu et al., 2006	White Leghorn female chickens	Cholesterol-free corn-soy diet	TRF (500 ppm)	Oral	4 weeks	↔	–	↓
			αTF (500 ppm)					
			αT3 (500 ppm)					
			γT3 (500 ppm)					
			δT3 (500 ppm)					
Qureshi et al., 2011	White Leghorn female chickens	Corn-soy diet	δT3 (50 ppm/kg diet)	Oral	4 weeks	↓	–	↓
**(B) MALE ANIMALS**								
Raederstorff et al., 2002	Male golden Syrian hamsters	Coconut-supplemented diet	γT3 (58 or 263 mg/kg/day)	Oral	4 weeks	–	–	↓
			Mixed T3 (263 mg/kg/day)					
Salman Khan et al., 2011	Hyperlipidemic male golden hamsters	LPS	Tocomin (10 mg/day)	Oral	10 days	–	–	↓
Qureshi et al., 2001a	Hereditary hypercholesterolemic swine	Corn-soybean diet	TRF (50 mg/kg diet)	Oral	6 weeks	–	↓	↓
			γT3 (50 mg/kg diet)					
Khor and Ng, 2000	Male golden Syrian hamsters	Semi-synthetic diet	αTF (30 ppm)	Oral	45 days	–	–	↑
Iqbal et al., 2003	Hypercholesterolemic female Sprague-Dawley rats	7,12-dimethylbenz [α]anthracene (DMBA)	Rice bran TRF (10 mg/kg/day)	Oral	6 months	–	–	↓
Minhajuddin et al., 2005	Male albino rats	Atherogenic diet	TRF (8 – 50 mg/kg)	Oral	1 week	–	–	↓
Zaiden et al., 2010	Hypercholesterolemic mice (LDLr^−/−^)	–	γδT3 (50 mg/kg)	Oral	4 weeks	↔	–	↓
Burdeos et al., 2012	Male F344 rats	High-fat (35%) diet	αTF (10 mg)	Oral	3 weeks	–	–	↔
			Rice bran TRF			↓	–	↓
Allen et al., 2015	Male Sprague-Dawley rats	Choline-deficient diet	αTF (200 mg/kg/day)	Oral	8 weeks	↔	↔	↔
Shibata et al., 2016	Male F344 rats	Western diet	αTF (50 mg/day)	Oral	3 weeks	–	↔	↔
			Rice bran T3 (11.1 mg/day)			–	↔	↓
			αTF (50 mg/day) + rice bran T3 (11.1 mg/day)			–	↔	↔

**Table 4:** The effects of vitamin E on metabolic parameters in hypertensive animal models.

**Researcher (Year)**	**Animal model**	**Types of diet/induction**	**Treatment and dose**	**Mode**	**Duration Weeks**	**Changes in metabolic parameters**
						**Hypertension**
Newaz et al., 2003	Male SHRs	Standard diet	γT3 (15, 30, 150 mg/kg diet)	Oral	12	↓
Miyamoto et al., 2009	Male SHRs	Standard diet	αTF (600 mg/kg diet)	Oral	12	↑

**Table 5:** The effects of vitamin E on metabolic parameters in MetS animal models.

**Researcher (Year)**	**Animal model**	**Types of diet/induction**	**Treatment and dose**	**Mode**	**Duration Weeks**	**Changes in metabolic parameters**			
						**Obesity**	**Hyperglycemia**	**Hypertension**	**Dyslipidemia**
Wong et al., 2012	Male Wistar rats	High-carbohydrate high-fat diet	Palm TRF (120 mg/kg/day)	Oral	8	↓	↓	↓	↓
Wong et al., 2015	Male Wistar rats	High-carbohydrate high-fat diet	αTF (85 mg/kg/day)	Oral	8	–	–	–	↓
			αT3 (85 mg/kg/day)			–	–	–	↓
			γT3 (85 mg/kg/day)			–	–	–	↓
			δT3 (85 mg/kg/day)			↓	↓	↓	↓

In the obese animal model, a study by Zhao et al. (2015) indicated that the marked increase in fasting blood glucose (FBG), insulin, fat content (mesenteric and epididymal), body weight, liver weight, and pro-inflammatory cytokines in high-fat diet-induced obese animals were efficiently counteracted by γT3 supplementation. An *in vivo* experiment by Alcala et al. (2015) demonstrated that obese mice fed on high-fat diet and supplemented twice a week with αTF (150 mg/kg) improved insulin sensitivity and hypertriglyceridemia, implicated through the reduction of oxidative stress and inflammatory response. On the contrary, vitamin E-enriched diet (2,000 IU/kg diet) (composition not mentioned) did not show any anti-atherogenic effects in leptin (ob/ob) and LDL receptor (LDLr^−/−^) deficient mice fed with high-fat diet. No significant reduction was observed in systemic oxidative stress, hyperlipidemia, and obesity in obese hyperlipidemic mouse model after 12 weeks of vitamin E intake (Hasty et al., 2007).

Drugs [streptozotocin (STZ)/nicotinamide], diet (e.g., high fructose/high fat) or combination of drugs and diet are the most common inducers of diabetes in animal models. Models using STZ develop type 1 diabetes mellitus (T1DM). T3 has been demonstrated to exert beneficial effects in T1DM model. Chou et al. (2009) reported that supplementation of 6 g/kg diet γT3 for 5 weeks raised insulin sensitivity, high density lipoprotein cholesterol (HDL-C), as well as lowered non-esterified fatty acid (NEFA), total cholesterol (TC):HDL-C ratio, and hepatic cholesterol concentration in diabetic animals. Kuhad et al. (2009) demonstrated that 10 weeks of 25, 50, or 100 mg/kg/day T3 (mixture of αT3, βT3, and‘ γT3) treatment dose-dependently ameliorated the adverse changes of body weight and glucose level in STZ-injected diabetic rats. Study by Kuhad and Chopra (2009a) compared the efficacy of 100 mg/kg αTF (commercially purchased) and 25, 50, or 100 mg/kg T3 (αT3: 14.6%, βT3: 1.2%, γT3: 17.4%, δT3:12.8%) in STZ-induced experimental diabetic animals. Supplementation of T3 gave a dose-dependent effect and more effective than αTF in improving body weight, glucose level, blood pressure (BP), food and water intake.

In addition, the hypoglycemic effects of tocotrienol-rich fraction (TRF) have been widely explored in animal studies. Budin et al. (2009) reported that palm TRF (composition not mentioned) significantly lowered serum glucose, glycated hemoglobin (HbA1c), TC, low density lipoprotein (LDL-C), and triglyceride (TG) levels, while increased levels of HDL-C in STZ-diabetic rats. The supplementation of palm TRF (αT3: 19.04%, βT3: 3.6%, γT3: 21.12%, δT3: 15%, αTF: 17.11%) has been further proven to reduce FBG levels in a recent study by Matough et al. (2014) using STZ-induced diabetic models. Instead of using a rat model, Fang et al. (2010) evaluated the effects of 50 mg/kg/day palm TRF (αT3: 23.54%, γT3: 43.16%, δT3: 9.83%, αTF: 23.5%) using diabetic C57BLKS/J-*Lepr Db/Db* mouse model. Administration of palm TRF was found to activate PPAR-α, PPAR-γ, and PPAR-δ to improve glucose utilization and insulin sensitivity in diabetic mice (Fang et al., 2010). Two different comparative animal studies were done by Siddiqui et al. on the anti-hyperglycemic effects of palm and rice bran TRF. In the earlier experiment, results showed that both 200 mg/kg/day rice bran TRF (αTF: 39.2%, αT3: 14.5%, βT3: 2.3%, γT3: 6.2%, δT3: 6.2%, unidentified T3: 31.6%) and palm TRF (αTF: 23%, αT3: 29%, γT3: 35%, δT3: 13%) ameliorated hyperglycemia-induced nephropathy through reduction of FBG and HbA1c, with palm TRF exerted greater efficiency (Siddiqui et al., 2010). In the latter experiment, treatment of 400 mg/kg/day rice bran TRF or 200 mg/kg/day palm TRF improved glycemic status and lipid profile, with more profound effects of palm TRF at lower dose compared to rice bran TRF at higher dose (Siddiqui et al., 2013).

Vitamin E has been extensively investigated for its anti-hyperlipidemic properties in various types of animals, such as chicken, hamsters, guinea pigs, swine, rats, and mice. Using female chickens as an animal model, Qureshi and Peterson (2001) showed that supplementation of 500 ppm rice bran TRF (αTF: 7%, αT3: 15%, γT3: 27%, δT3: 8%, *d*-P_21_-T3: 12%, *d*-P_25_-T3: 15%, unidentified T3: 11%) for 4 weeks significantly reduced TC, LDL-C, apolipoprotein B (apoB), and TG levels in these animals. However, no changes were observed in HDL-C and apolipoprotein A1 (apoA1) levels. Yu et al. (2006) compared the anti-hypercholesterolemic effects between TRF (αTF: 16.8%, αT3: 26.5%, γT3: 42.5%, δT3: 14.1%), αTF, αT3, γT3, and δT3 at the dose of 500 ppm in healthy female chickens fed with cholesterol-free corn-soy diet. The findings displayed the comparative potential of cholesterol reduction properties in ascending order of αTF < αT3 or TRF < γT3 < δT3. A few years later, Qureshi et al. (2011) demonstrated that addition of δT3 into chicken feed (50 ppm/kg diet) significantly lowered body weight, TC, LDL-C, and TG levels of female chickens.

For experiments using male hamster model, Raederstorff et al. (2002) reported that dietary feeding of γT3 or mixed T3 [αT3: 29.5%, βT3: 3.3%, γT3: 41.4%, δT3: 0.1%, others (mainly γTF): 25.1%] yielded lower levels of TC and LDL-C but did not alter TG and HDL-C levels, whereby γT3 had more potent effect than T3 mixture. Salman Khan et al. (2011) further reported that 10 mg/day tocomin (αT3: 6.4%, βT3: 1%, γT3: 10.2%, δT3: 3.2%, αTF: 5.7%) significantly lowered levels of TG, TC, very low density lipoprotein cholesterol (VLDL-C), LDL-C, LDL-apoB, sd-LDL-C, sd-LDL-apoB, free and esterified cholesterol, but increased levels of lb-LDL-C and lb-LDL-apoB in male hyperlipidemic hamsters induced by bacterial lipopolysaccharide (LPS). For study using hereditary hypercholesterolemic swine model, Qureshi et al. (2001a) reported the beneficial effects of 50 mg/kg diet TRF (αTF: 4.5%, αT3: 15.7%, βT3: 2.3%, γT3: 34.6%, δTF: 4.8%, δT3: 7.9%, *d*-P_21_-T3: 13.7%, *d*-P_25_-T3: 14.4%, unidentified T3: 2.1%) and γT3 in lowering TC, LDL-C, apoB, TG, glucose, and glucagon levels, while increasing insulin level. A more comprehensive animal study was done by Khor and Ng (2000) to compare the actions of vitamin E on 3-hydroxy-3-methylglutaryl-CoA (HMG-CoA) reductase activity using hamsters and guinea pigs. Male golden Syrian hamsters supplemented with αTF for 45 days displayed hypercholesterolemic effects which raised TC, LDL-C, and TG levels but no changes in HDL-C level. Meanwhile, male albino guinea pigs were intraperitoneally administered with different doses of αTF and T3 (αT3: 23.3%, γT3: 50.8%, δT3: 24.6%, αTF: 0.2%, γTF: 1.1%) for 6 consecutive days. Administration of T3 alone exhibited highest inhibition of HMG-CoA reductase activity (48%), followed by combination of αTF and T3 (35%). For treatment of αTF alone, higher doses of αTF resulted in stimulation of HMG-CoA reductase activity. These results suggested that αTF is hypercholesterolemic and attenuated the effects of T3 in HMG-CoA reductase inhibition. The findings from these various types of animal model indicated the potential of vitamin E (T3 had greater efficiency than TF) as anti-hyperlipidemic agent in both male and female animals.

**Figure 1:**
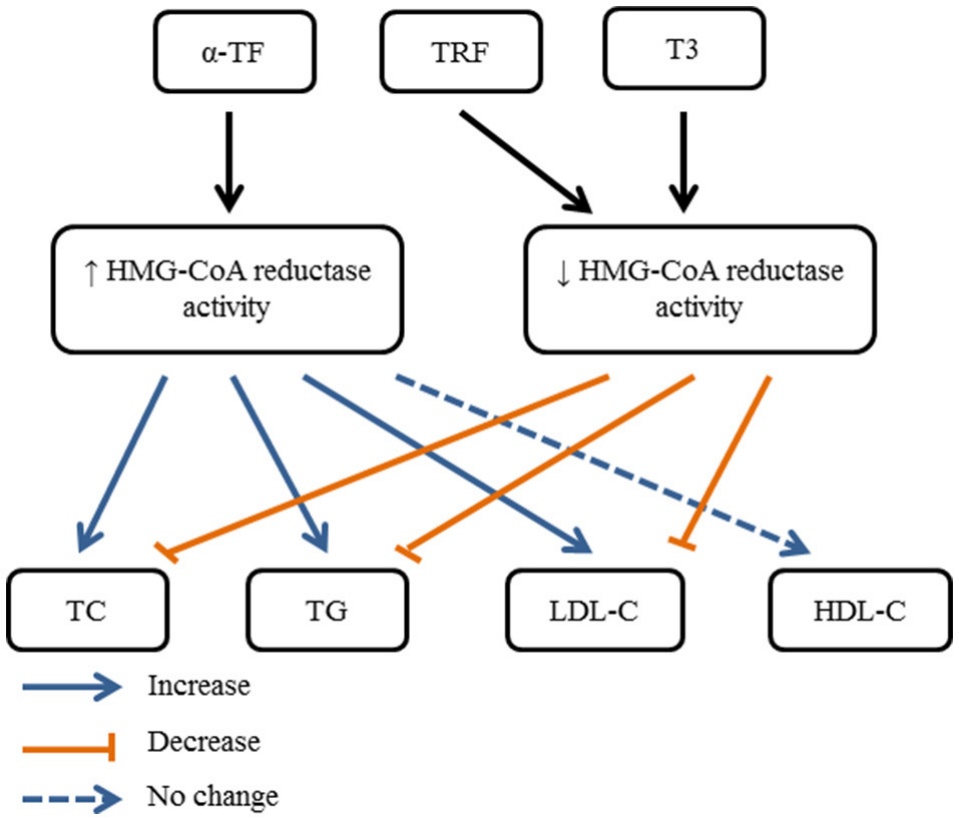
Summary of possible hypocholesterolemic and hypercholesterolemic actions of tocopherol and tocotrienol, mediated through activation or inhibition of HMG-CoA reductase activity.

Among those rat and mouse models, Iqbal et al. (2003) demonstrated a chemical carcinogen-induced hypercholesterolemic rat model using 7,12-dimethylbenz[α]anthracene (DMBA), which introduced mammary carcinogenesis and hypercholesterolemia in rats. Treatment of 10 mg/kg/day rice bran TRF (composition not mentioned) for 6 months reversed DMBA-induced hyperlipidemia (decrease in TC and LDL-C levels) through suppression of enzymatic activity and protein mass of HMG-CoA reductase. Minhajuddin et al. (2005) also demonstrated significant decline in TG, TC, LDL-C levels and inhibited HMG-CoA reductase activity in male albino rats fed with atherogenic diet and treated with TRF (αT3: 14.6%, βT3: 2.2%, γT3: 6.2%, δT3: 6.2%, αTF: 14.7%, βTF: 10.6%, γTF: 3.1%, δTF: 10.7%, unidentified T3 and TF: 31.7%) for 1week. In the subsequent year, Zaiden et al. (2010) found that 4 weeks of γδT3 (50 mg/kg) treatment caused significant reduction in TC, TG, and LDL-C, but not in HDL-C levels. On the other hand, Burdeos et al. (2012) showed that rice bran TRF (αT3: 31.4%, γT3: 50.5%, δT3: 0.4%, αTF: 1.9%, γTF: 2.1%, δTF: 2.0%) ameliorated high-fat diet-mediated dyslipidemia (lowering of body weight, mesenteric fat, epididymal fat, plasma and hepatic TG) with concurrent reduction of oxidative stress markers in liver and plasma. However, αTF supplemented group showed no significant difference in total lipids of liver and plasma. Furthermore, Allen et al. (2015) demonstrated that supplementation of αTF (200 mg/kg/day) was not able to improve any metabolic changes but further increased hepatic TG level. Shibata et al. (2016) further supported that feeding of αTF (50 mg/day) was not sufficient to exhibit anti-hyperlipidemic effects on Western diet-fed rats as rice bran T3 (αT3: 0.2 mg, γT3: 9.5 mg, δT3: 0.3 mg). The combination of αTF and rice bran T3 also suppressed the anti-hyperlipidemic effects of rice bran T3 supplementation alone. The hypocholesterolemic and hypercholesterolemic actions of tocopherol and tocotrienol are illustrated in Figure 1.

A few research articles presented the heterogeneous effects of vitamin E in hypertensive laboratory animal models. Newaz et al. (2003) showed that the intake of γT3 (15, 30, 150 mg/kg) added into the standard diet for 3 months significantly improved nitric oxide synthase (NOS) activity and increased plasma nitrite level thus leading to a reduction in systolic BP of spontaneously hypertensive rats (SHRs). Interestingly, an experiment designated by Miyamoto et al. (2009) found that addition of high content of αTF (600 mg/kg) into standard diet increased systolic BP of SHRs.

For MetS animal model, there were few animal studies investigated the effects of vitamin E on metabolic parameters. Palm TRF (αTF: 22.9%, αT3: 31.9%, βT3: 2.1%, γT3: 24.8%, δT3: 18.3%) has been reported by Wong et al. (2012) to induce metabolic changes in rats fed with high-carbohydrate high-fat diet. The metabolic changes include the reduction of abdominal circumference, omental fat, FBG, systolic BP, TG, NEFA, and the elevation of lean mass and food intake. After a few years, Wong et al. (2015) compared the potential of αTF, αT3, γT3, and δT3 at the dosage of 85 mg/kg/day in improving metabolic conditions. The findings showed the superiority of δT3 among the treatments in minimizing liver injuries, reducing total fat mass, abdominal circumference, adiposity index, retroperitoneal fat, epididymal fat, FBG, systolic BP, TC, NEFA, and TG levels.

Even though the vitamin E mixtures used in these *in vivo* studies differs in the effective doses, composition, and treatment duration, the findings indicated the obvious beneficial effects of T3 in preventing MetS components in general. Tocotrienol improved insulin sensitivity, glucose tolerance, lipid profile, reduced BP, and fat content. The beneficial properties are mainly mediated through the reduction of oxidative stress (Kuhad and Chopra, 2009a; Siddiqui et al., 2010) and inflammatory response (Siddiqui et al., 2013; Alcala et al., 2015; Zhao et al., 2015). For evaluation on the effects of TF, combination of TF and T3 was demonstrated to inhibit the bioactivity of T3. However, a low dose of TF seems to be beneficial in ameliorating the metabolic disease conditions (Newaz et al., 2003; Miyamoto et al., 2009). Hence, it is revealed that vitamin E showed potential effects in preventing MetS which T3 may provide more promising effects compared to TF based on preclinical studies.

## The protective properties of vitamin E in human studies

Vitamin E is a minor but essential nutrient for health, which is ingested with unsaturated fat-containing foods. The Recommended Dietary Allowance (RDA) of vitamin E is equivalent to 35 μmol/day (15 mg/day) for both men and women (Food Nutrition Board, 2000). The inadequacy of vitamin E (plasma αTF level <12 μmol/L) in human is commonly related with multiple health complications such as increased infection, anemia, hindering of growth, as well as unhealthy infant and mother during pregnancy (Traber, 2014). A recent study found that patients with MetS had impaired absorption of dietary vitamin E as lower levels were detected in MetS subjects compared to healthy adults (Mah et al., 2015). The bioavailability of αTF was unaffected by dairy fat ingestion. This is probably due to the inhibition of αTF absorption in the small intestine and impairment of αTF transport by the occurrence of higher inflammatory response and oxidative stress in this condition (Mah et al., 2015). Vitamin E has been reported to possess anti-inflammatory (Siddiqui et al., 2013; Heng et al., 2015), anti-oxidative (Kuhad and Chopra, 2009b; Siddiqui et al., 2010), and anti-hypercholesterolemic (Yu et al., 2006; Salman Khan et al., 2011) properties via regulation of various signaling pathways. Considering MetS is a disease comprises of induction of inflammatory response and oxidative stress, thus vitamin E may have the potential on MetS.

### Vitamin E and MetS

The association between vitamin E supplementation and MetS has been investigated in a study among MetS subjects who were non-vitamin and non-anti-oxidant users (*n* = 80). Participants were randomly allocated with αTF (800 mg/day), γTF (800 mg/day), combination of αTF and γTF (800 mg each/day), or placebo for 6 weeks. Combination of αTF and γTF showed superiority in reducing lipid peroxides, tumor necrosis factor-alpha (TNF-α), malondialdehyde (MDA), 4-hydroxynonenal (HNE), and high sensitivity C-reactive protein (hs-CRP) levels, suggesting the potential in ameliorating oxidative stress, nitrative stress, and inflammatory response in MetS patients (Devaraj et al., 2008). In addition, a more recent randomized, double blind, placebo-controlled trial involving adults with MetS (*n* = 57) aged 20–60 years showed supplementation of mixed T3 (400 mg/day) for 16 weeks exerted beneficial effects in chronic inflammation [reduced interleukin-6 (IL-6) and TNF-α] and lipid profiles (reduced TC, LDL-C, and HDL-C) in adults with MetS as compared to baseline (Heng et al., 2015). These aforementioned findings are still inadequate to be conclusive on the potential of vitamin E. Here, we comprehensively collate the evidence on the effects of vitamin E on each of MetS features in human studies to further evaluate its potential as therapeutic agent for MetS.

### Vitamin E and obesity

Evidence on the direct effect of vitamin E on body weight reduction in humans is still not available. However, a cross-sectional study demonstrated an inverse relationship between obesity and serum vitamin E level, which was conducted in morbidly obese patients (*n* = 110) and healthy subjects (*n* = 56) aged 19–59 years not taking any multivitamin supplements. Morbidly obese men and women had significantly lower levels of serum αTF compared to healthy control (Aasheim et al., 2008). Besides, the effect of vitamin E on obesity-related complications has been reported in a recent human study. Irandoost et al. (2013) examined the effects of grape seed oil (containing high amounts of TF and T3) (exact composition not mentioned) consumption on inflammation and insulin resistance in overweight or obese females (*n* = 44) aged 20–50 years. Treatment with grape seed oil for 8 weeks improved inflammatory condition, evident by reduced hs-CRP and TNF-α. In addition, grape seed oil reduced homeostatic model assessment of insulin resistance (HOMA-IR) scores, which indicated the improvement of insulin resistance in obese females.

### Vitamin E and diabetes

Diabetes is a complication often accompanied with increased oxidative stress, whereby vitamin E has protective property against lipid peroxidation (reviewed by Pazdro and Burgess, 2010). The effects of vitamin E in diabetic patients have been extensively investigated. A cohort study was carried out between 1967 and 1972 to evaluate the ability of antioxidant intake to predict type II diabetes among Finnish men (*n* = 2,285) and women (*n* = 2,019) aged 40–69 years. The subjects were free of diabetes or heart disease at baseline and with reported daily energy intake of 800–6,000 kcal through a dietary history interview. During a 23-year follow-up, a total of 164 males and 219 females were involved. Results from the cohort indicated that intakes of αTF (RR: 0.66, 95% CI: 0.49–0.90), γTF (RR: 0.77, 95% CI: 0.57–1.03), δTF (RR: 0.69, 95% CI: 0.51–0.93), and βT3 (RR: 0.76, 95% CI: 0.56–1.03) were significantly associated with reduced risk of diabetes (Montonen et al., 2004). However, findings from another cohort study implemented few years later by Kataja-Tuomola et al. (2011) found that no association was detected between dietary supplementation of αTF (RR: 0.92, 95% CI: 0.71–1.19), βTF (RR: 1.06, 95% CI: 0.82–1.36), and βT3 (RR: 1.04, 95% CI: 0.80–1.35) and risk of diabetes among male smokers (*n* = 25,505) aged 50–69 years.

In addition, a double-blind, placebo-controlled trial was conducted to investigate the impact of vitamin E (αTF or mixed TF rich in γTF) in type II diabetes patients (*n* = 55, mean age: 61.3 years). Results revealed that pure αTF reduced plasma F_2_-isoprostanes (lipid peroxidation marker) while mixed TF reduced both plasma F_2_-isoprostanes and neutrophil leukotriene B_4_ (inflammation marker) in patients with type II diabetes (Wu et al., 2007). Next, a prospective clinical study was conducted to evaluate the role of vitamin E (composition not mentioned) therapy as primary prophylaxis and secondary intervention in type I and II diabetic patients. The study indicated promising results as vitamin E supplementation was able to delay the onset of diabetic complications (decreased post-prandial blood glucose), slow down the progression of the complications, reduce diastolic BP, and lower TC levels (Baburao Jain and Anand Jain, 2012). A randomized, double blinded, placebo-controlled trial was performed by Haghighat et al. (2014) to evaluate the effects of T3 on microalbuminuria, inflammation, and nitrosative stress in type II diabetes patients (*n* = 50) aged 35–60 years. The results showed daily administration of palm-based T3 (mixture of 38.4% total T3, 13.2% αT3, 16.6% γT3, and 16% αTF) with added canola oil for 8 weeks decreased proteinuria (reduced urine microalbumin) and protected kidney against inflammation (reduced hs-CRP); but caused no significant changes in nitric oxide (NO) and creatinine levels. Another similar double-blind, placebo-controlled, randomized trial was undertaken among patients with type II diabetes mellitus (*n* = 50, aged 35–60 years) treated by non-insulin hypoglycemic drugs. Patients in the intervention group were assigned to receive 15 ml of T3 (200 mg/day) enriched canola oil for 8 weeks. Results demonstrated that T3-enriched canola oil significantly reduced FBG and MDA while total antioxidant capacity was increased. No significant effect was observed on fasting insulin concentration and HOMA-IR scores (Vafa et al., 2015).

### Vitamin E and dyslipidemia

Lipid imbalance [REFERRING to elevated TG and decreased HDL-C] is the hallmark of dyslipidemia, which predisposes an individual to cardiovascular disease. Effects of vitamin E on dyslipidemia in human studies were contradictory. A study by Qureshi et al. (2001b) demonstrated that the combination of TRF and lovastatin was more effective in lowering TC and LDL-C levels among subjects supplemented with American Heart Association (AHA) Step-1 diet compared to individual treatment. Qureshi et al. (2002) recruited hypercholesterolemic subjects (*n* = 90) with serum cholesterol levels >5.7 mmol/L. Results showed a dose-dependent effect of TRF (25, 50, 100, or 200 mg/day) to decrease TC, LDL-C, apoB, and TG levels compared to baseline. In the subsequent year, Baliarsingh et al. (2005) performed a randomized, double blind, placebo controlled trial to investigate the therapeutic effects of rice bran TRF (approximately 7.5% αTF, 4.6% δTF, 14.6% αT3, 2.2% βT3, 38.8 γT3, 29.9% δT3, and 2.4% unidentified T3) on dyslipidemia in type II diabetic patients (*n* = 19) but free from other illnesses. Administration of TRF (3 mg/kg) for 60 days was useful for prevention and treatment for hyperlipidemia as TRF significantly reduced serum total lipids (21%), TC (28%), and LDL-C (38%) levels of type II diabetic patients. However, no significant changes were observed in TG, HDL-C, and VLDL-C levels. Apart from that, a randomized controlled study recruited healthy older individuals within two age groups: 35–49 years (*n* = 31) and >50 years (*n* = 31) to determine the effects of 6 months treatment with 160 mg/day TRF (containing 70.4 mg αT3, 4.8 mg βT3, 57.6 mg γT3, 33.6 mg δT3, and 48 mg αTF) supplementation on lipid profile and oxidative status. Healthy individuals (aged >50 years) supplemented with TRF had higher level of HDL-C, higher gluthathione peroxidase (GPx) activity, lower protein carbonyl contents, lower level of advance glycated end products (AGEs), and lower superoxide dismutase (SOD) activity after 6 months compared to placebo group (Chin et al., 2011). A randomized, double-blind, placebo-controlled, parallel trial on patients undergoing chronic hemodialysis (*n* = 81) was performed. The supplementation of TRF (consisting of 180 mg/day T3 and 40 mg/day TF) for 16 weeks improved lipid profiles (reduced TC, reduced cholesteryl-ester transfer protein activity, increased HDL-C, and increased apoA1) when compared with placebo (Daud et al., 2013). Mustad et al. (2002) investigated the effects of 200 mg/day T3 supplementation with different compositions (αT3+γT3, high γT3, or P25-complex T3) for 4 weeks on total lipids, FBG, and oxidative stress markers (8-*iso*-prostaglandin F_2α_) in healthy hypercholesterolemic individuals (*n* = 67). Results indicated negligible effects of T3 supplementation on lipids, glucose, and 8-*iso*-prostaglandin F_2α_ levels in this double-blind, randomized, parallel-design study. Moreover, hypercholesterolemic subjects (*n* = 19) with BMI >25 and aged 25–55 years were enrolled to determine the suppression of serum lipids by γδT3 in a clinical trial. Results demonstrated that 8 weeks of γδT3 treatment (120 mg/day) lowered levels of serum TG, VLDL-C, and chylomicrons; while levels of TC, LDL-C, and HDL-C remained unchanged in the treated group compared to placebo (Zaiden et al., 2010).

Negative outcome was detected in a double-blind placebo controlled study involving hypercholesterolemic population (*n* = 51) given 4 weeks of low-fat diet, followed by supplementation of α-, γ-, or δ-tocotrienyl acetate (150 mg/day) for 8 weeks. Tocotrienol supplementation had no effects on lowering of LDL-C and apoB levels in the population (O'Byrne et al., 2000). Next, Rasool et al. (2006) reported no beneficial effects of 2 months T3-rich vitamin E (80, 160, or 320 mg/day) supplementation in reducing serum TC and LDL-C concentrations in healthy male subjects (*n* = 36).

### Vitamin E and hypertension

Oxidative stress is a key factor in the pathogenesis of hypertension (Rodrigo et al., 2011; Montezano and Touyz, 2012). In a randomized double-blind placebo-controlled clinical trial, the role of palm oil TRF in preventing pregnancy-induced hypertension was investigated among healthy primigravidae (*n* = 299). Daily supplementation of 100 mg TRF (composition not mentioned) from early second trimester until delivery reduced the incidence of pregnancy-induced hypertension (Mahdy et al., 2013). Besides, the effect of TF on BP has been investigated in type II diabetic patients (*n* = 58). Surprisingly, daily treatment with 500 mg/day αTF or mixed TF (composed of 60% δT3, 25% δT3, and 15% αTF mixture) for 6 weeks significantly increased systolic BP, diastolic BP, pulse pressure and heart rate compared to placebo (Ward et al., 2007).

As summary, literature on the direct effects of vitamin E in reducing abdominal circumference, body weight or fat mass was absent. Nonetheless, vitamin E improved inflammatory status and insulin resistance in obese patients, which were closely related to the occurrence of MetS. For diabetes patients, all studies indicated that vitamin E (either individual or mixtures of vitamin E isomers) is beneficial in reducing blood glucose, inflammation, lipid peroxidation, BP, and TC levels. Additionally, vitamin E provided positive outcome in most of the studies on dyslipidemia including patients with hypercholesterolemia, diabetes, undergoing chronic hemodialysis, and healthy individuals. However, data on the effects of vitamin E in hypertensive patients are limited with heterogeneous findings. Human studies displayed inconsistent findings which may be due to the different composition, doses, administration frequency, and duration of the treatment. The distribution and metabolism of vitamin E may also differ between humans. These reported literature clearly elucidated that the beneficial effects of vitamin E outweighed the adverse effects in humans. However, more clinical trials are required to further confirm the effects of vitamin E in humans. Vitamin E appears to be a promising agent as treatment for MetS through the findings from both *in vivo* and human studies.

## Comparison between the effects of tocopherol and tocotrienol

Inflammation and oxidative stress play a crucial role in MetS. Obesity and dyslipidemia induce abnormal growth of adipose tissue, contributing to the overproduction of inflammatory mediators [particularly TNF-α, IL-6, and interleukin-1 beta (IL-1β)] and activation of nuclear factor-kappa B (NF-κB) (Weng-Yew and Brown, 2011). Hyperglycemia leads to increased production of AGEs, which enhance the synthesis of pro-inflammatory cytokines in monocytes and macrophages. Additionally, high glucose levels stimulate production of superoxide anion (${\text{O}}_{2}^{-}$) and reactive oxygen species (ROS) through activation of glycolysis (Gonzalez et al., 2012). During hypertension, an inverse correlation will be observed between BP with SOD and GPx activity, causing the increase in oxidative stress (Baradaran et al., 2014). It has been widely reported that inflammation and oxidative stress are indeed closely related to MetS, therefore agents possessing both anti-inflammatory and anti-oxidative properties should have potential in regulating MetS. This idea stimulated research on the health benefits of vitamin E. Recent preclinical animal studies as well as human observational studies and clinical trials clearly indicated the dissimilarity in biological properties between TF and T3.

Tocopherol appears to be a potent natural anti-oxidant via its capability to utilize the free hydroxyl group on the chromanol ring to capture free radicals (Engin, 2009). A low dose of αTF (150 mg/kg) given twice a week showed its capability in reducing production of hydroxyl radical and scavenging peroxides to improve insulin sensitivity and reduce TG level (Alcala et al., 2015). Among the isomers of TF, combination of αTF and γTF showed better effects than the supplementation of αTF or γTF alone in reducing oxidative stress in MetS (Devaraj et al., 2008) and diabetic (Wu et al., 2007) patients. Nonetheless, Miyamoto and co-workers reported that a relatively high dose of αTF intake (600 mg/kg diet) had detrimental effects on stroke-prone SHRs. Treatment with αTF elevated BP and lipid peroxide concentrations in serum and brain tissue of SHRs (Miyamoto et al., 2009). Thus, high-dose αTF can act as pro-oxidant in the body, thereby damaging the organs.

Data on the anti-oxidative activity over the past two decades showed that T3 is a stronger class of antioxidant than TF (Serbinova et al., 1991; Serbinova and Packer, 1994; Kamal-Eldin and Appelqvist, 1996). Recent study demonstrated that 100 mg/kg of T3 had greater efficacy as compared to αTF in decreasing lipid peroxidation [lowered thiobarbituric acid-reactive substance (TBARS) levels] and oxidative stress (increased non-protein thiols, SOD activity, catalase and reduced NO levels) (Kuhad and Chopra, 2009a). Apart from that, 200 mg/kg palm TRF was more effective than rice bran TRF in improving anti-oxidant enzyme activity and inhibiting lipid peroxidation through decreasing NO, TBARS, and MDA level while increasing catalase, GPx, gluthathione reductase, and SOD activity (Siddiqui et al., 2010). The ratio of TF:T3 content in rice bran and palm TRF was 39:61 and 23:77 respectively. Supplementation of rice bran T3 also reduced phospholipid hydroperoxides (PLOOH) in the liver of high-fat diet-induced hyperlipidemic rats. Meanwhile, supplementation of αTF did not show any effects in reducing PLOOH levels (Burdeos et al., 2012). Based on recent animal studies involving T3 or TRF rich in T3, findings displayed promising effects of T3 as an anti-oxidant in ameliorating metabolic conditions. Tocotrienol is able to (a) reduce BP through increasing nitrite levels and NOS activity (Newaz et al., 2003); (b) improve glycemic status and lipid profile via the increase of SOD and GPx activity while lowering of MDA and HNE thus reducing deoxyribonucleic acid (DNA) damage (Budin et al., 2009; Matough et al., 2014); (c) reduce TG level leading to the attenuation of TG-induced PLOOH accumulation (Asai et al., 1999; Burdeos et al., 2012).

*In vivo* studies showed that the beneficial effects of T3 in metabolic regulation were elicited through the reduction in inflammatory response. Treatment of T3 for 10 weeks inhibited the elevation of TNF-α, transforming growth factor-beta (TGF-β), and IL-1β, which are the modulators for inhibition of NF-S signaling pathway and apoptosis (reduced caspase-3) in STZ-induced diabetic rats. Treatment of αTF showed similar potential but with less effectiveness (Kuhad and Chopra, 2009a; Kuhad et al., 2009). The anti-hyperlipidemic effect of δT3 was also observed through the reduction of TNF-α (83%) in the serum of 5-week-old healthy female chickens (Qureshi et al., 2011). Siddiqui et al. (2013) compared the effects of rice bran TRF (400 mg/kg/day) and palm TRF (200 mg/kg/day) supplementation in the down-regulation of TGF-β expression, fibronectin, and collagen type IV in the kidneys of STZ-induced diabetic rats, with palm TRF (lower dose) showing higher efficacy than rice bran TRF (higher dose). Findings in the study done by Zhao et al. (2015) further supported previous findings on the effects of T3 as an anti-inflammatory agent. Tocotrienol treatment was able to inhibit systemic and adipose tissue inflammation. Administration of γT3 reduced IL-6, IL-1β, and monocyte chemoattractant protein-1 (MCP-1), indicating that γT3 treatment led to a reduction in the recruitment of adipose tissue macrophages (ATMs). On the other hand, administration of αTF managed to improve inflammatory status, characterized by the inhibition of p38 phosphorylation to basal level, decreased levels of IL-6, TNF-α, vand plasminogen activator inhibitor-1 (PAI-1) (Alcala et al., 2015). In a human study, combination of αTF and γTF showed better effects than the supplementation of αTF or γTF alone in reducing inflammation markers in MetS subjects (Devaraj et al., 2008). However, supplementation of either αTF or γTF (500 mg/day) was unlikely to reduce inflammation in diabetic patients (Wu et al., 2007).

Based on a plethora of accumulated evidence, T3 had greater efficacy as an anti-hypercholesterolemic agent compared to αTF (Yu et al., 2006). Shibata et al. (2016) reported for the first time that αTF exerted an interfering effect on the anti-hyperlipidemic effects of T3 through suppression of T3-induced reduction of TG and TC as well as attenuation of T3-induced increases in expression of *Cpt-1a* and *Cyp7a1*. Cholesterol is excreted in the form of bile acids. Upregulation of *Cpt-1a* and *Cyp7a1* induced by T3 were closely related with reduced plasma TG and increased bile acid synthesis from cholesterol. Apart from that, there was an inverse relationship between the dosage of αTF and its cholesterol-lowering potential (Alcala et al., 2015; Allen et al., 2015). The uniqueness of T3 having a shorter, double-bond, farnesylated tail which is responsible for its hypocholesterolemic action. Tocotrienol acts as an inhibitor for HMG-CoA reductase (Salman Khan et al., 2011), an important enzyme for cholesterol synthesis in the mevalonate pathway. Inhibition of HMG-CoA reductase prevents the production of mevalonate in the cascade that eventually synthesized cholesterol. On the contrary, the longer, single-bond, phytyl tail of TF failed to exert this function (Sen et al., 2006).

The bioavailability of both TF and T3 differs in terms of the absorption due to the shorter and thicker tail of T3, allowing them to move throughout the entire cell with more flexibility (Levine, 2008). However, many studies also indicated that existence of TF inhibited T3 uptake in peripheral tissues, which has been explained through several potential mechanisms. Firstly, the preferential binding of αTF to α-tocopherol transfer protein (α-TTP) compared to other isoforms of TF and T3 facilitates the transfer of αTF to tissues (Hosomi et al., 1997; Uchida et al., 2012). Secondly, αTF induced changes in membrane permeability thus suppressing the cellular uptake of T3 (Shibata et al., 2010). Additionally, T3 has been reported to undergo a series of bio-conversions through C-methylation and reduction, leading to the production of αTF as the end product (Qureshi et al., 2015).

Overall, T3 was found to be more effective compared to TF as anti-oxidative, anti-inflammatory, and anti-hypercholesterolemic agent. Furthermore, these findings further supported by studies indicating that palm TRF had greater efficacy compared to rice bran TRF, as rice bran TRF had lesser content of T3. Whereas for TF, combination of TF isomers was found to be more beneficial compared to the effects of individual TF. A lower dose of TF has been reported to provide positive outcome but higher dose of TF was detrimental. Therefore, it can be postulated that higher content of T3 plays a crucial role in ameliorating metabolic complications.

## Conclusion and future direction

Researchers' attention on vitamin E has been shifted from TF to T3 since the discovery of the cholesterol-lowering (Qureshi et al., 1986) and anti-cancer (Sundram et al., 1989) properties of T3 several decades ago. Notably, the current documented evidence indicated that the intake of naturally occurring vitamin E as dietary supplement favors the prevention or treatment of MetS, with T3 being more superior than TF. In other words, vitamin E appears to be a potential current medication for MetS as the advantage of vitamin E (particularly T3) in treating most of the clinical conditions associated with MetS. In fact, the composition of TF and T3 need to be carefully considered in order to avoid interference and adverse effects of high-dose TF. More importantly, possible adjunct prevention or treatment using vitamin E will be safe, efficacious, and highly cost effective to substitute the current available pharmacological drugs. Future research on the therapeutic potential of vitamin E with particular emphasis on understanding the mechanistic basis and the signaling pathways involved will be a necessity to better predict the beneficial effects of vitamin E.

## Author contributions

SKW performed literature search and drafted the manuscript. K-YC, FHS, FA, and SI-N provided critical review for the manuscript. SI-N gave the final approval for the publication of this manuscript.

### Conflict of interest statement

The authors declare that the research was conducted in the absence of any commercial or financial relationships that could be construed as a potential conflict of interest.

## Abbreviations

α-TTP: α-tocopherol transfer protein
AHA: American Heart Association
AGEs: advance glycated end products
apoA1: apolipoprotein A1
apoB: apolipoprotein B
ATMs: adipose tissue macrophages
BP: blood pressure
*d*-P_21_-T3: *d*-desmethyl tocotrienol
*d*-P_25_-T3: *d*-didesmethyl tocotrienol
DMBA: 7,12-dimethylbenz [α]anthracene
DNA: deoxyribonucleic acid
FBG: fasting blood glucose
GPx: gluthathione peroxidase
HbA1c: glycated hemoglobin
HDL-C: high density lipoprotein cholesterol
HMG-CoA: 3-hydroxy-3-methylglutaryl-CoA
HNE: hydroxynonenal
HOMA-IR: homeostatic model assessment of insulin resistance
hs-CRP: high sensitivity C-reactive protein
IDL: intermediate-density lipoprotein
IL-6: interleukin-6
IL-1β: interleukin-1 beta
lb-LDL-C: large buoyant low density lipoprotein cholesterol
LDL-C: low density lipoprotein cholesterol
LPS: lipopolysaccharides
MCP-1: monocyte chemoattractant protein-1
MDA: malondealdehyde
MetS: metabolic syndrome
NEFA: non-esterified *fatty acid;*
NF-κB: nuclear factor-kappa B
NO: nitric oxide
NOS: nitric oxide synthase
${\text{O}}_{2}^{-}$: superoxide anion
PAI-1: plasminogen activator inhibitor-1
PLOOH: phospholipid hydroperoxides
PPAR: peroxisome proliferator-activated receptor
RDA: recommended dietary allowance
ROS: reactive oxygen species
RR: risk ratio
sd-LDL-C: small density low density lipoprotein cholesterol
SHRs: spontaneous hypertensive rats
SOD: superoxide dismutase
STZ: streptozotocin
TF: tocopherol
T3: tocotrienol
TBARS: thiobarbituric acid reactive substances
TC: total cholesterol
TG: triglyceride
TGF-β: transforming growth factor-beta
TNF-α: tumor necrosis factor-α
TRF: tocotrienol rich fraction
VLDL-C: very low density lipoprotein cholesterol
↑: significant increase
↓: significant decrease
↔: no effect.
